# High Performance 0D ZnO Quantum Dot/2D (PEA)_2_PbI_4_ Nanosheet Hybrid Photodetectors Fabricated via a Facile Antisolvent Method

**DOI:** 10.3390/nano12234217

**Published:** 2022-11-27

**Authors:** Shijie Liu, Hao Li, Haifei Lu, Yanran Wang, Xiaoyan Wen, Shuo Deng, Ming-Yu Li, Sisi Liu, Cong Wang, Xiao Li

**Affiliations:** 1 School of Science, Wuhan University of Technology, Wuhan 430070, China; 2 School of Electronics and Information Engineering, Harbin Institute of Technology, Harbin 150001, China; 3 Hisense Visual Technology Co., Ltd., Qingdao 266555, China

**Keywords:** 2D perovskite, ZnO quantum dot, antisolvent method, photodetector, outstanding stability

## Abstract

Two-dimensional (2D) organic−inorganic perovskites have great potential for the fabrication of next-generation photodetectors owing to their outstanding optoelectronic features, but their utilization has encountered a bottleneck in anisotropic carrier transportation induced by the unfavorable continuity of the thin films. We propose a facile approach for the fabrication of 0D ZnO quantum dot (QD)/2D (PEA)_2_PbI_4_ nanosheet hybrid photodetectors under the atmospheric conditions associated with the ZnO QD chloroform antisolvent. Profiting from the antisolvent, the uniform morphology of the perovskite thin films is obtained owing to the significantly accelerated nucleation site formation and grain growth rates, and ZnO QDs homogeneously decorate the surface of (PEA)_2_PbI_4_ nanosheets, which spontaneously passivate the defects on perovskites and enhance the carrier separation by the well-matched band structure. By varying the ZnO QD concentration, the Ion/Ioff ratio of the photodetectors radically elevates from 78.3 to 1040, and a 12-fold increase in the normalized detectivity is simultaneously observed. In addition, the agglomeration of perovskite grains is governed by the annealing temperature, and the photodetector fabricated at a relatively low temperature of 120 °C exhibits excellent stability after a 50-cycle test in the air condition without any encapsulation.

## 1. Introduction

Photodetection provides a feasible approach for interpreting the unreadable optical information by converting it into electrical signals, which has been sparking burgeoning interests for widespread applications, such as optical telecommunication, environmental monitoring, biomedical imaging, distributing sensor networks, and the internet of things [1,2,3,4]. The rapid expansion of information technology has been fueling the demand for high-performance photodetectors with a high signal-to-noise ratio, sensitivity, response speed, spectrum selectivity, and stability, which were proposed as “5S” figure-of-merits for the evaluation of photodetection capability [5]. Perovskites have emerged as promising photoactive materials with a combination of advantages over conventional semiconductors, including easy solution processability, a large absorption coefficient, a long carrier diffusion length, and high carrier mobility [6,7]. Inheriting from the excellent photoelectric capacities of their three-dimensional (3D) counterparts, 2D perovskites have been recently developed with a considerably improved atmospheric stability owing to the protection of hydrophobic organic chain spacers enclosed in [MX_6_]^4−^ metal halide octahedra [8], which is believed to be a savior for long-term stable perovskite-based photodetection [9]. As of now, research on the 2D perovskite-based photodetectors is still in the early embryonic stage, and their application suffers from a huge hindrance in the fabrication of continuous thin films from isolated nanosheets [10]. Recently, the one-step antisolvent method has been reported as a successful attempt for manufacturing high quality (PEA)_2_PbI_4_ thin films at relatively low temperatures owing to the accelerated nucleation and grain growth governed by polarity and saturated vapor pressure [11]. As a typical Ruddlesden—Popper (RP) phase perovskite, the Van der Waals interactions between layers within (PEA)_2_PbI_4_ nanosheets can restrict the charge-carrier transportation along out-of-plane direction [12], which remains a critical challenge of the undesirable performance in comparison to that of 3D perovskite photodetectors.

Perovskite photoactive thin films have been successfully fabricated via various approaches, i.e., chemical vapor deposition [13], mechanical exfoliation [14], electrochemical deposition [15,16,17], and spinning coating [18]. Alternative fabrication techniques allow the construction of heterostructures by introducing foreign low-dimensional materials, offering an efficient route for improving the optoelectronic response of the resulting perovskite thin films. For instance, the Cs_2_AgBiBr_6_/WS_2_/graphene vertical heterostructure photodetectors demonstrated orientationally intensified charge transfer [19]. In addition, direct incorporation of low-dimensional materials with a high conductivity (i.e., graphene QDs [20] and MXene nanosheets [21]) can well balance the simplicity of fabrication and efficient charge transfer, which is commonly accompanied with inevitably knotty issues of the extra noise in dark and lattice miss-match in the hybrid photoactive layers. Meanwhile, ZnO QDs possess various advantages, including large resistance in the dark, high electron mobility, and a wurtzite structure [22], and the improved crystallization of CsPbBr_3_ thin films was observed by blending with ZnO QDs [23], providing an adequate filler for the fabrication of high-performance photodetectors. In this work, we demonstrate novel 0D ZnO QD/2D (PEA)_2_PbI_4_ nanosheet hybrid photodetectors fabricated via a facile antisolvent method under atmospheric conditions. The homogeneous decoration of ZnO DQs on a 2D (PEA)_2_PbI_4_ nanosheet surface instantaneously realizes the defect passivation and construction of carrier transfer paths within the photoactive layers. With a well-balanced ZnO concentration, the normalized detectivity (D^*^) of the 0D/2D hybrid perovskite photodetector radically increases to 15.40 × 10^9^ Jones with an excellent I_on_/I_off_ ratio of 1040. Temperature effect on the performance of photodetectors is attributed to the morphological evolution of the hybrid perovskite thin films, and superior stability of the device is observed within 50 test cycles, offering an auspicious way to overcome the breakthrough of 2D perovskite photodetectors for the “5S” requirements.

## 2. Materials and Methods

**Chemicals and Reagents:** Zinc acetate dihydrate (C_4_H_6_O_4_Zn·2H_2_O, 99.0%; Sinopharm Group Chemical Reagent Co., Ltd. A. R, Shanghai, China), potassium hydroxide (KOH, 85%; Aladdin, Shanghai, China), methanol (CH_3_OH, 99.5%; Sinopharm Group Chemical Reagent Co., Ltd. A. R, Shanghai, China), chloroform (CH_3_Cl, 99.0%; Sinopharm Group Chemical Reagent Co., Ltd. A. R, Shanghai, China), phenethylammonium iodide (PEAI, 99.5%; Xi’an Polymer Light Co., Xi’an, China), lead iodide (PbI_2_, 99.0%; Sinopharm Group Chemical Reagent Co., Ltd. A. R, Shanghai, China), and N, N-Dimethylformamide (DMF, 99.0%; Sinopharm Group Chemical Reagent Co., Ltd. A. R, Shanghai, China). All chemical reagents mentioned above were used directly, without any further purification.

**Material Synthesis:** The ZnO quantum dots (QDs) were prepared through an optimized solvothermal method as reported in our previous work [24]. Specifically, 1.9582 g of zinc acetate dihydrate was dissolved in 46 mL of methanol and heated up to 60 °C. Meanwhile, 0.8528 g of KOH was dissolved in 46 mL of methanol and dropped into the zinc acetate dihydrate methanol solution for 8–12 min. The mixed solution kept stirring for 2.15 h under 60 °C, and was washed twice by the precipitation method. After synthesis, the resulting ZnO QDs were dispersed in 6 mL of chloroform. The (PEA)_2_PbI_4_ precursors were prepared in the atmosphere at room temperature. Specifically, the mixture of 199.2 mg PEAI and 184.0 mg PbI_2_ was dissolved in 1 mL of DMF, and the solution was treated with ultrasonication for 20 minutes at room temperature.

**Device Fabrication:** Prior to spin-coating, each glass substrate with a size of 2.5 × 2.5 cm^2^ was ultrasonicated in anhydrous ethanol (99.0%) and acetone (99%) for 20 minutes, respectively. Subsequently, 100 μL of (PEA)_2_PbI_4_ precursor solution was initially dropped on the glass substrate, and 100 μL of chloroform antisolvents with various contents of ZnO QDs: 0 mg/mL (PD1), 0.032 mg/mL (PD2), 0.048 mg/mL (PD3), 0.097 mg/mL (PD4), 0.194 mg/mL (PD5), 0.388 mg/mL (PD6), and 0.776 mg/mL (PD7). After incubation for 1 min, the photoactive thin films were spin-coated layer-by-layer at 800 rpm for 20 s and 2000 rpm for 30 s, respectively. After coating 3 layers, the samples were annealed at 120 °C for 1 min between spin-coating for each layer and were finally treated with a 10 min annealing at an identical temperature to improve crystallization. To investigate the annealing temperature effect, the samples fabricated with a 0.097 mg/mL of chloroform antisolvent were annealed on a hot plate in the atmosphere at room temperature (PD8), 80 °C (PD9), 100 °C (PD10), and 140 °C (PD11). Finally, a pair of 80 nm-thick Au electrodes with a spacing distance of 200 μM was deposited via thermal evaporation.

**Device Characterization:** The morphologies of ZnO QD-decorated (PEA)_2_PbI_4_ nanosheets were characterized by a transmission electron microscope (TEM, JEM-1400 Plus, JEOL, Tokyo, Japan), and scanning electron microscope (SEM) images of each sample were obtained by a Regulus8100 system (Hitachi, Tokyo, Japan). The elemental analysis of the samples was carried out by an energy dispersive X-ray spectroscopy system (EDS, Regulus8100, Hitachi, Tokyo, Japan), and the surface morphological characterization was realized with atomic force microscopy (AFM, Dimension Icon, Bruker, Karlsruhe, Germany). The crystallinity of the samples was analyzed by X-ray diffractometer (XRD, D8 Advance, Bruker, Karlsruhe, Germany; D/MAX-RB, Rigaku, Tokyo, Japan). The absorption spectra were recorded on a UV/vis spectrophotometer (UV-2600, Shimadzu, Kyoto, Japan), and the steady-state photoluminescence (PL) spectra were required by a spectrofluorophotometer (RF-6000, Shimadzu, Kyoto, Japan) with an excitation light of 350 nm. The time-resolved PL (TRPL) spectra of (PEA)_2_PbI_4_ thin films were recorded with a spectrofluorometer (Fluo Time 300, PicoQuant, Berlin, Germany) with a pumping laser of 375 nm and a probing laser of 526 nm. The photoelectric performances of all devices were measured with a semiconductor characterization system (4200, Keithley, Cleveland, OH, USA), and a monochrome adjustable light source (CME-OPS1000, Microenerg, Beijing, China) was engaged as an illumination source.

## 3. Results and Discussion

**Figure 1:**
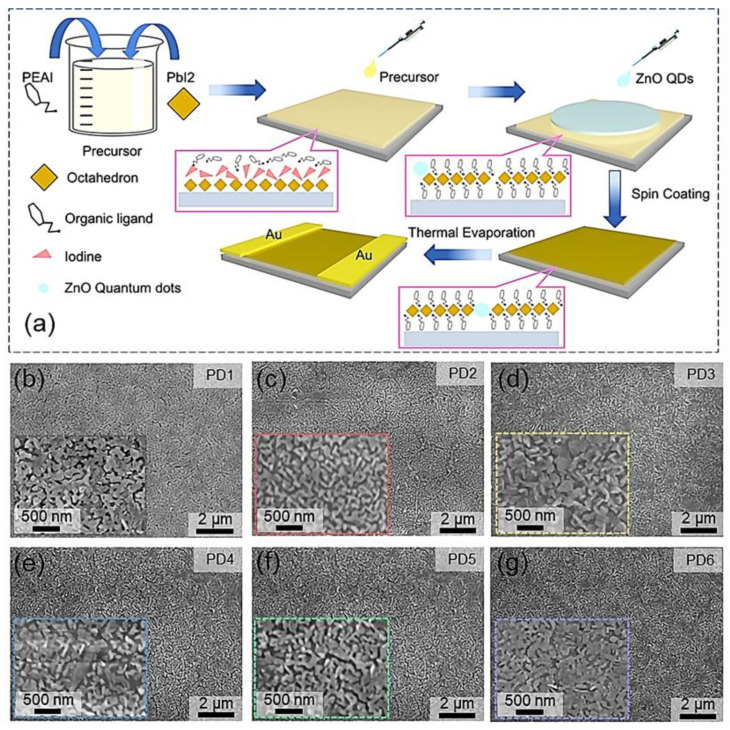
(**a**) Schematic diagram of the fabrication process of ZnO QD decorated (PEA)_2_PBI_4_ nanosheet photodetectors via antisolvent method. (**b**–**g**) Scanning electron microscope (SEM) images of the ZnO QD/(PEA)_2_PBI_4_ nanosheet samples with various proportions of ZnO QD: PD1 (0 mg/mL), PD2 (0.032 mg/mL), PD3 (0.048 mg/mL), PD4 (0.097 mg/mL), PD5 (0.194 mg/mL), and PD6 (0.388 mg/mL).

Figure 1 shows the effect of ZnO QD concentrations on the morphological evolution of the zero-dimensional (0D) ZnO QD and 2D (PEA)_2_PbI_4_ nanosheet hybrid thin films. The fabrication process of ZnO QD/(PEA)_2_PbI_4_ nanosheet photodetectors is depicted in Figure 1a. Briefly, the ZnO QD/(PEA)_2_PbI_4_ thin films were fabricated by blending ZnO QD chloroform solution with various concentrations after dropping 100 μL PEAI and PbI_2_ mixture on glass substrate. According to previous works, highly crystallized 2D perovskite nanosheet thin films with a continuous morphology could be hardly achieved [25], and an effective approach for improving crystallinity was reported by adding antisolvents during thin film growth due to the accelerated nucleation site formation and grain growth rates [26]. Providing the distinctive polarity (μ_C_ = 1.06 D) of chloroform over that of DMF (μ_D_ = 3.82 D) [11], the nucleation site formation and grain growth rates were significantly boosted, and thus, the packed morphology of the pristine (PEA)_2_PbI_4_ nanosheet thin films was obtained, as shown in Figure 1b. As shown in Figure S1a, the ZnO QDs are well-decorated on the surface of the (PEA)_2_PbI_4_ nanosheet with an average diameter of ~6.22 nm after blending into the (PEA)_2_PbI_4_ nanosheet precursor solution. In addition, (PEA)_2_PbI_4_ nanosheets generally crystallized in the monoclinic space group C2/m with lattice parameters a = 3.25 nm, b = 0.61 nm, and c = 0.62 nm [27], and wurtzite ZnO with relatively smaller lattice parameters a = b = 0.32 nm and c = 0.52 nm [28], allowing preferential adsorption of ZnO QDs on (PEA)_2_PbI_4_ nanosheets. As a result, ZnO QDs were evenly decorated on the (PEA)_2_PbI_4_ nanosheets, as clearly witnessed in Figure S1b, and bright diffraction spots of (200) and (020) planes [29] in the selected area electron diffraction (SAED) pattern evidenced the existence of (PEA)_2_PbI_4_ in the mixed solution, as shown in Figure S1c. As shown in Figure 1 and Figure S1d, the packed and continuous surface morphology was comparably observed for each ZnO QD/(PEA)_2_PbI_4_ nanosheet sample, with a variation of ZnO QD concentrations between 0 and 0.776 mg/mL. Meanwhile, the neglectable effect of ZnO QDs on the surface morphology can also be witnessed with a similar root-mean-squared roughness (RRMS) for the devices blending with various concentrations of 0 mg/mL (14.4 nm) and 0.097 mg/mL (17.1 nm), as shown in Figure S1e–f.

**Figure 2:**
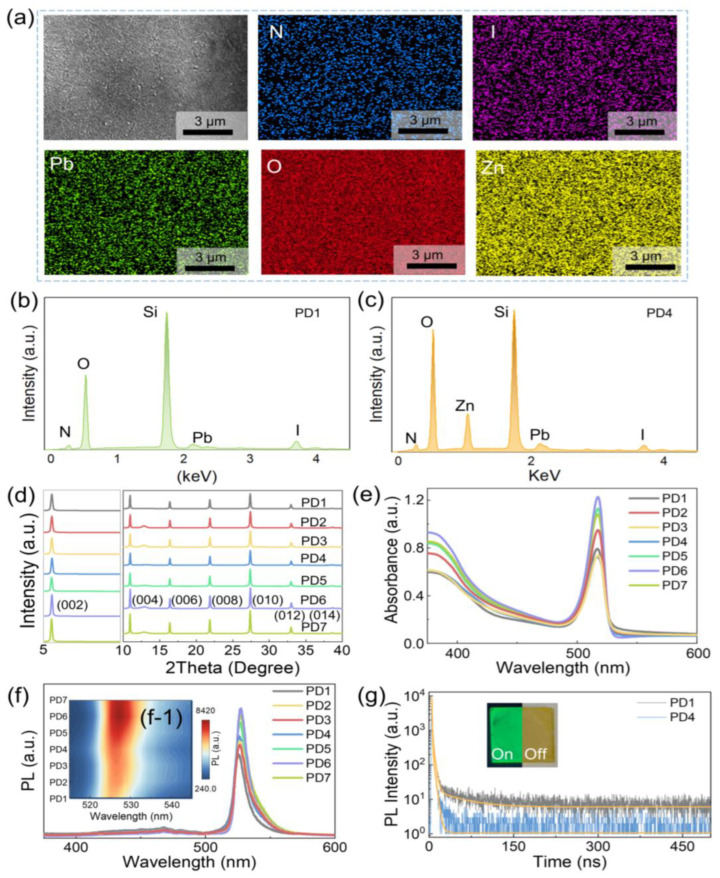
(**a**) Energy dispersive X-ray spectroscopy (EDS) element maps of the photodetector PD4. Energy dispersive X-ray spectroscopy (EDS) spectra of the photodetectors (**b**) PD1 and (**c**) PD4. (**d**) Corresponding X-ray diffraction (XRD) spectra. (**e**) Absorbance and (**f**) room-temperature PL spectra of the devices. (**f**–**1**) The contour maps of wavelength dependent PL for the devices. (**g**) Time-resolved photoluminescence (TRPL) spectra of the photodetectors PD1 and PD4. (Inset) The optical images of the sample under UV illumination (left) and white light (right).

To further investigate the effect of ZnO QD, the developments of optical properties and crystal structures were systematically investigated with diverse concentrations between 0 and 0.776 mg/mL, as shown in Figure 2. As shown in Figure 2a, the elements N, I, Pb, O, and Zn were evenly observed throughout the whole surface of the PD4 device fabricated with 0.097 mg/mL ZnO QD antisolvent solution, indicating a uniform distribution of ZnO QD in the hybrid thin films. In terms of the elementary composition of the samples, the existence of (PEA)_2_PbI_4_ was verified with the occurrence of the Kα_1_ peak at 0.392 keV of N, Mα_1_ peak at 2.346 keV of Pb, and Lα_1_ peak at 3.94 keV of I regardless of the ZnO QD concentration [30], and the Lβ_1_ peak at 1.035 keV of Zn [31] can only be witnessed with the hybrid thin films blending with 0.097 mg/mL ZnO QDs, as shown in Figure 2b,c. For each sample, the characteristic peaks at 5.5°, 10.9°, 16.4°, 21.9°, 27.4°, 33.0°, and 38.7° can be assigned to the periodical diffraction peaks of (001) series of reflections ((00x), x = 2, 4, 6), (010), (012), and (014) peaks [32], as shown in Figure 2d. Practically, (002) peaks strikingly appeared in comparison with other peaks, manifesting a preferential crystallization of the thin films [33]. As revealed in Figure 2e, the noticeable exciton absorption peak of (PEA)_2_PbI_4_ [34] was constantly observed at 517 nm in the absorbance spectrum for each sample, suggesting an identical bandgap of ~2.364 eV the hybrid thin films irrespective of the ZnO QD concentration as conformed with the Tauc plots shown in Figure S2. Correspondingly, the near band emission for each sample was similarly witnessed at 526 nm without any shifting as exhibited with room temperature photoluminescence (PL) spectra in Figure 2f, and the peak intensity gradually elevated along with the increased ZnO QD concentration as observed in Figure 2f, which can be possibly induced by intensified irradiative combination with the reduced defect state density due to the surface passivation with ZnO QDs [35]. To verify the hypothesis, time-resolved photoluminescence (TRPL) spectra of the photodetectors fabricated without (PD1) and with ZnO QD (PD4) were characterized, as shown in Figure 2g. The TRPL curves were well fitted by a multiexponential function with three decay components τ_1_ (the excitation relaxation), τ_2_ (the interaction between excitons and phonons), and τ_3_ (the recombination of excitons with defects) [36]:

(1) $$I\left(t\right)=A_{1}exp\left(-t/\tau_{1}\right)+A_{2}exp\left(-t/\tau_{2}\right)+A_{3}exp\left(-t/\tau_{3}\right)$$

where *A*_1_, *A*_2_, and *A*_3_ denote the proportionality factor for each decay period. The average decay lifetime (τ_avg_) of the device can be given with [37]:

(2) $$\tau_{Avg}=A_{1}\times \tau_{1}+A_{2}\times \tau_{2}+A_{3}\times \tau_{3}$$

As summarized in Table 1, the τ_1_ and τ_2_ were comparable for the devices, and the significant decrease in τ_avg_ from 5.16 ns (PD1) to 0.94 ns (PD4) was primarily ascribed to the restricted recombination of excitons with defects [38], which can be beneficial for the carrier transportation within the photodetectors. As shown in the inset in Figure 2g, the continuous morphology of the thin films was observed with the PD4 device, and strong green irradiation under UV light illumination verified the high quality of the spin-coated thin films.

**Figure 3:**
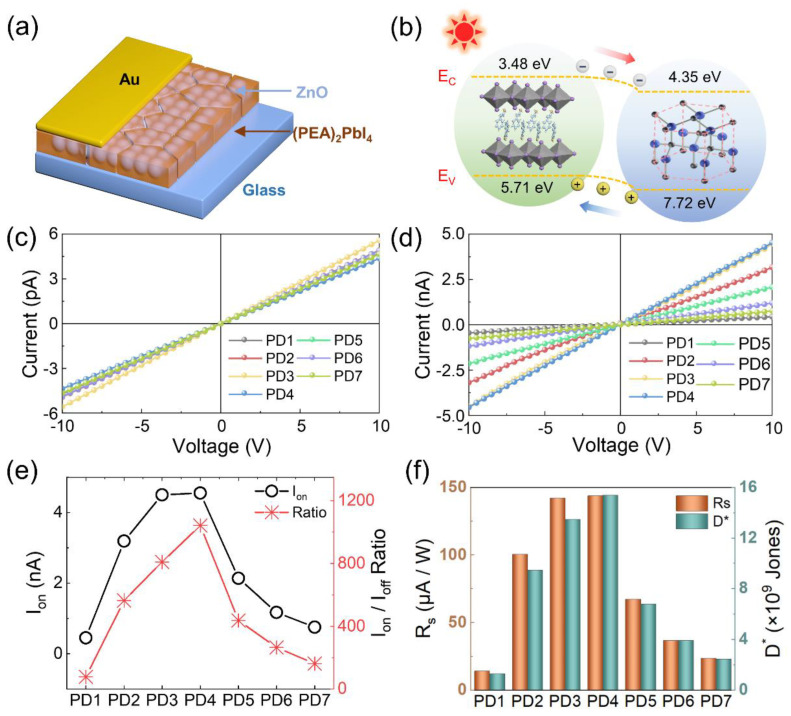
The photoelectric performance of the devices. (**a**) Scheme of the configuration and (**b**) the energy-band diagram of the ZnO QD/(PEA)_2_PBI_4_ nanosheet photodetector. I-V characteristics of the devices (**c**) in the dark (I_off_) and (**d**) under illumination (I_on_) of 500 nm UV light (1.97 mW cm^−2^). (**e**) The I_on_ and I_on_/I_off_ ratio of each device. (**f**) Responsivity (R_s_) and normalized detectivity (D *) of each device with different contents of ZnO QDs.

Figure 3 shows the evolution of photoelectric performance of the hybrid ZnO QD/(PEA)_2_PbI_4_ nanosheet photodetectors as a function of ZnO QD concentrations, and the configuration of the device is depicted in Figure 3a. In order to deeply study the role of ZnO QD, the energy band diagram of the device is shown in Figure 3b. The conduction band minimum (E_C_) and valence band maximum (E_V_) of ZnO are 4.35 and 7.72 eV [39], and (PEA)_2_PbI_4_ possesses relatively higher E_C_ (3.48 eV) and E_V_ (5.71 eV) [40]. Thus, enhanced carrier separation can be correspondingly expected from (PEA)_2_PbI_4_ nanosheets to ZnO QDs under light illumination, overcoming the hindrance of charge transfer between isolated nanosheets within the thin films. As revealed in Figure 3c, the current in dark (I_off_) of devices linearly elevated depending on the bias voltage without any barrier formation, and I_off_ was comparable among photodetectors, suggesting that incorporation of ZnO QD would not introduce extra noise. As shown in Figure 3d, the elevated photocurrent (I_on_) was observed at each voltage with the increased ZnO QD concentrations from 0 to 0.097 mg/mL, resulting in a noticeable increase in I_on_ from 0.46 to 4.55 nA at 10 V under 500 nm illumination owing to improved carrier transportation induced by the efficient defect passivation and photoexcited electron separation. With the further increased concentration, the I_on_ gradually deteriorated to 0.76 nA when the concentration reached 0.776 mg/mL, as shown in Figure 3e, which can because the formation of undesirable ion scattering sites as a result of the aggregation of exceeded ZnO QDs at grain boundaries of (PEA)_2_PbI_4_ severely hindered the carrier transportation [41]. Correspondingly, the I_on_/I_off_ ratio, an important metric to evaluate the antinoise capacity of photodetectors, radically increased by 13-folds from 78.3 to 1040 in comparison with the pristine device, as shown in Table 2. The responsivity (R_s_) can be expressed as [42]:

(3) $$R_{S}=\frac{I_{\mathrm{on}}-I_{\mathrm{off}}}{P\times S}$$

where *P* represents the light intensity (1.97 mW cm^−2^), and S is the active area between the two electrodes (0.016 cm^−2^). As shown in Figure 3f, the R_s_ is developed as a function of the ZnO QD concentrations, leading to the optimized R_s_ of 143.94 μA W^−1^ for the PD4 device. Given that the shot noise generally determines the total noise in photodetectors, the normalized detectivity (D^*^) becomes a crucial metric for the evaluation of the devices, which can be given with [43]:

(4) $$D^{*}=\frac{I_{\mathrm{on}}-I_{\mathrm{off}}}{P}\times \sqrt{\frac{1}{2\times e\times S\times I_{\mathrm{off}}}}$$

where *e* denotes the elementary charge (1.6 × 10^−19^ C). In contrast to the pristine device (PD1), the D^*^ of the PD4 device obviously elevated from 1.31 × 10^9^ to 1.54 × 10^10^ Jones, as shown in Figure 3f, and the D^*^ gradually decreased along with the further increased ZnO QD concentration due to the strictly deteriorated I_on_. The conversion efficiency of incident photons into electrons for photodetectors can be determined by the external quantum efficiency (EQE) [44]:

(5) $$EQE=\frac{hc}{e\lambda }\times R_{s}\times 100\%$$

where *h*, *c,* and λ represent the Planck’s constant, the velocity of light in a vacuum, and the wavelength of irradiated light, respectively. Similar to R_s_, the EQE markedly boosted from 0.0035% to 0.0357% when the ZnO QD concentration increased from 0 to 0.097 mg/mL, and the deterioration in EQE was observed with further increased concentration as evidenced with Figure S3a.

**Figure 4:**
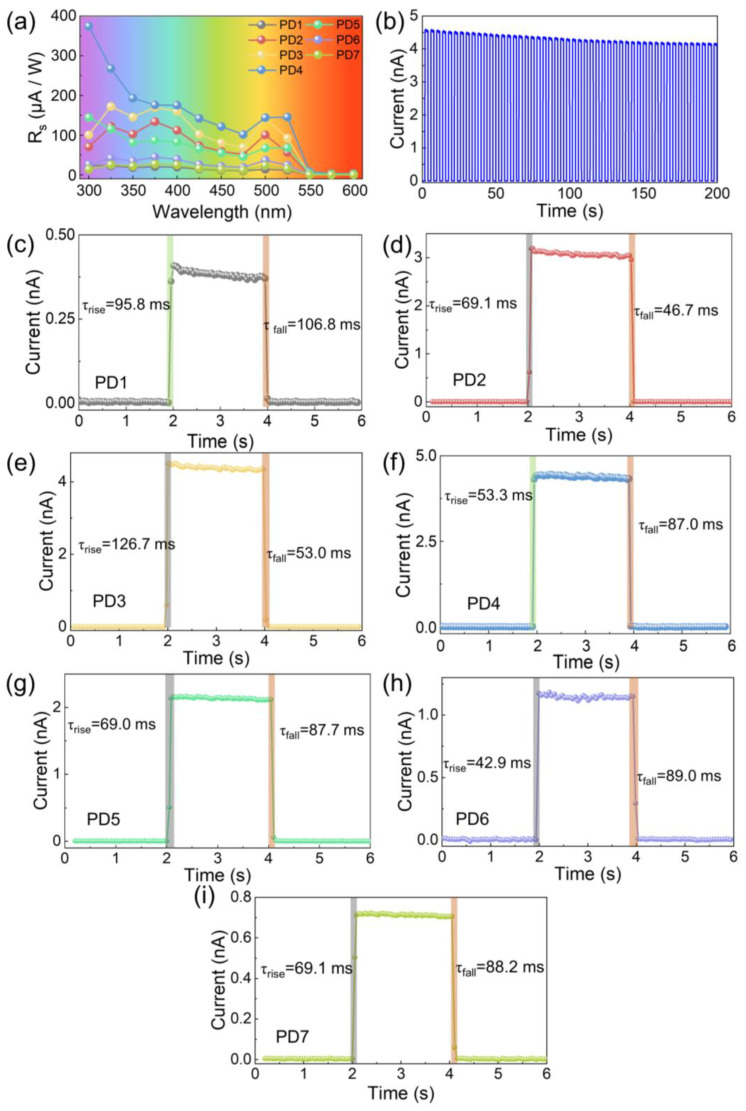
(**a**) The responsivity (R_s_) of the photodetectors within a broadband spectrum wavelength. (**b**) Transient response of the device PD4 within multiple test cycles. Single-period response of the photodetectors (**c**) PD1, (**d**) PD2, (**e**) PD3, (**f**) PD4, (**g**) PD5, (**h**) PD6, and (**i**) PD7 under 500 nm light irradiation at 10 V bias voltage.

The development of photoelectric performance for the photodetectors depending on the ZnO QD concentrations was further investigated at various spectrum wavelengths from 300 to 600 nm and testing periods, as shown in Figure 4. As observed in Figure 4a, the effect of ZnO QD concentrations on the R_s_ was consistently witnessed throughout the whole response spectra between 300 and 550 nm, and the R_s_ of the device PD4 was consequently superior to the photodetectors at each wavelength. Particularly, the optimized R_s_ was obtained at 500 nm for most devices, and the R_s_ of the devices PD4 (374.57 μA W^−1^) at 300 nm was evidently higher than that at 500 nm (143.94 μA W^−1^), which can be ascribed to the additional photogenerated carriers from ZnO QDs under UV illumination. As a result, the similar behaviors of D^*^ and EQE were observed depending on the variation of spectrum wavelengths, as shown in Figure S3b,c, indicating that blending with ZnO QDs can be an advantageous strategy for the improved performance for the 2D perovskite photodetectors over a broad wavelength range. As shown in Figure S3d, the I_on_/I_off_ ratio of the device PD4 was always higher than that of the device PD1, and the decreased ratios below 375 nm were derived from the unfavorable excitation at much lower light intensities. The photodetector PD4 remained at excellent stability with a negligible deterioration after a continuous test of 50 cycles in the air, as evidenced in Figure 4b. The τ_rise_ (the period for the current elevated from 10% to 90% of the I_on_) and τ_fall_ (the period for the current declined from 90% to 10% of the I_on_) of each photodetector was within 150 ms, as shown in Figure 4c–i. Compared with the pristine device, the photodetectors fabricated with ZnO QDs exhibited faster τ_rise_ and τ_fall_, suggesting accelerated carrier mobility within the hybrid thin films. 

**Figure 5:**
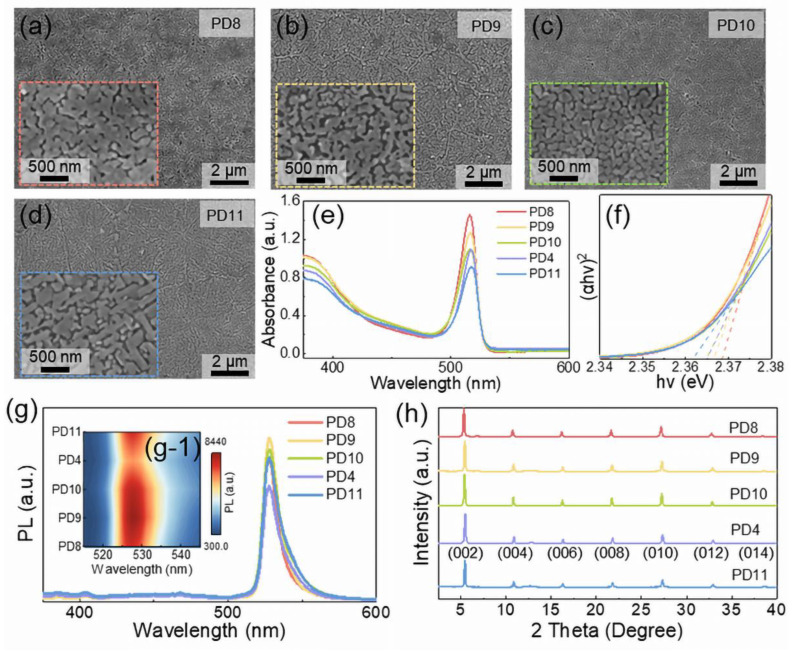
SEM images of the ZnO QD/(PEA)_2_PBI_4_ nanosheet devices fabricated with a 0.097 mg/mL ZnO QD chloroform antisolvent at different annealing temperatures: (**a**) room temperature (PD8), (**b**) 80 °C (PD9), (**c**) 100 °C (PD10), and (**d**) 140 °C (PD11). (**e**) Absorbance spectra and (**f**) Tauc plots of the samples. (**g**) The PL spectra and (**g-1**) the contour maps of wavelength-dependent PL for the devices. (**h**) XRD spectra of each sample.

The evolution of the morphological and optical characteristics of ZnO QD/(PEA)_2_PbI_4_ nanosheet hybrid thin films fabricated with an identical ZnO QD concentration of 0.097 mg/mL was investigated with a variation of annealing temperatures from room temperature to 140 °C, as shown in Figure 5. As shown in Figure 5a–c, the uniform and dense morphology was evenly observed at each temperature, and agglomeration gradually occurred along with the increased temperature, which can be beneficial for the crystallization of the hybrid perovskite thin films [45,46,47]. Above 120 °C, the severe truncation was observed between grains, as shown in Figure 5d, which can be induced by the partially melt crystallization of (PEA)_2_PbI_4_ [48]. As shown in Figure 5e, there was a slight red shift of the exciton absorption peak as a function of annealing temperature, and thus, the bandgap gradually decreased from 2.368 eV to 2.362 eV due to the expansion of grain size [49], as shown in Figure 5f. As observed in Figure 5g, the PL peak initially descended depending on the annealing temperature induced by the intensified surface scattering along with morphological development, and peak intensity slightly ascended above 120 °C as a result of decreased roughness along with the melt crystallization [50]. In addition, the characteristic peaks were similarly observed without any shift depending on annealing temperatures, and the (002) peak increased after annealing in comparison with the thin films fabricated at room temperature, suggesting a preferential crystallization along [002], as evidenced in Figure 5h.

**Figure 6:**
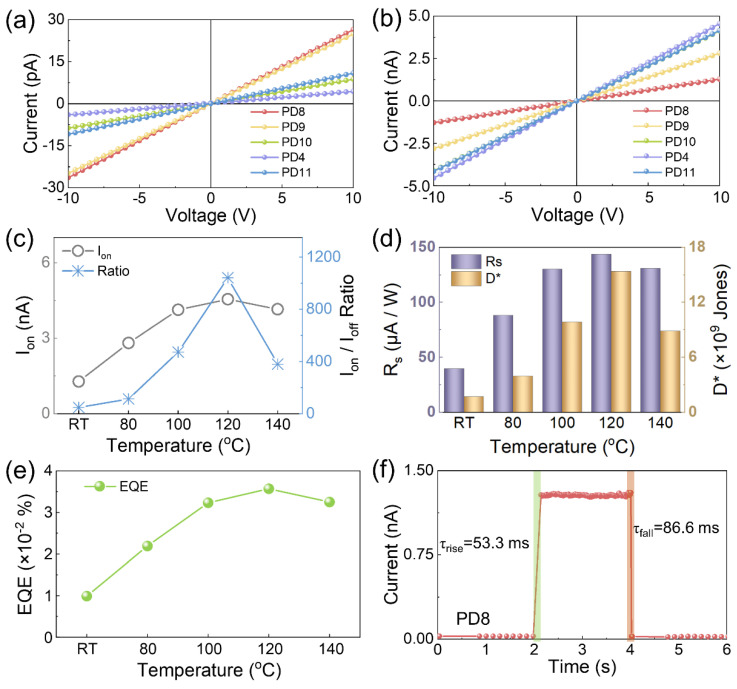
The photoelectric performance of the photodetectors fabricated at various annealing temperatures. I-V characteristics of the devices (**a**) in the dark and (**b**) irradiated under 500 nm UV light (1.97 mW cm^−2^). (**c**) The plots of I_on_ and I_on_/I_off_ ratio for each device. (**d**) R_s_, D*, and (**e**) external quantum efficiency (EQE) of each device fabricated at distinct temperatures. (**f**) The transient response within a single period for the photodetector PD.

**Figure 7:**
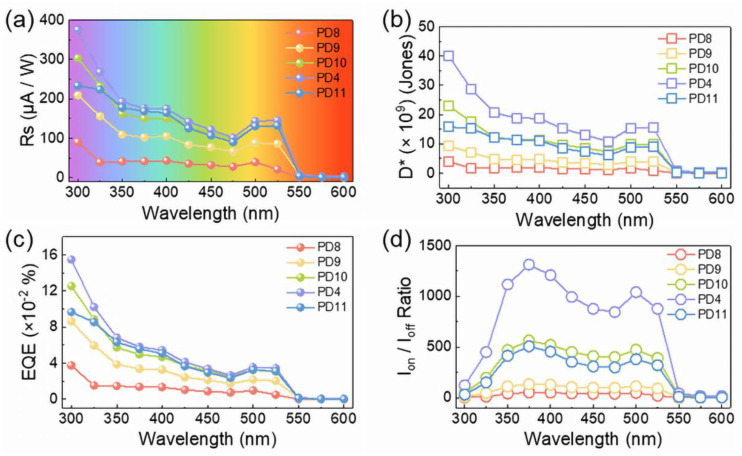
The spectrum dependent (**a**) responsivity (R_s_), (**b**) normalized detectivity (D*), (**c**) external quantum efficiency (EQE), (**d**) I_on_/I_off_ ratio plots with different annealing temperature.

The temperature effect on the photoelectric performance of the ZnO QD/(PEA)_2_PbI_4_ nanosheet hybrid photoreactors is shown in Figure 6. As shown in Figure 6a, the I_off_ was slightly higher at relatively lower temperatures within the whole bias range due to the denser morphology, and superior I_on_ was constantly obtained with the PD4 device at each bias as observed in Figure 6b, manifesting a sufficient temperature for the high crystallinity (PEA)2PbI4 thin films at 120 °C. Therefore, the Ion at 10 V was elevated by 3.6-folds from room temperature to 120 °C, and the Ion/Ioff ratio correspondingly increased by 21.4-folds as shown in Figure 6c. Depending on the annealing temperature, a comparable trend was witnessed with the Rs, D^*^, and EQE, as shown in Figure 6d–e. The increase was likewise observed for each metric as a function of temperature, and slight decreases occurred above 120 °C. As evidenced in Figure 6f and Figure S4, the response time was similar for the devices fabricated at various temperatures, suggesting that the ZnO concentration played a more critical role in the response speed than the annealing temperature. As shown in Figure 7, the photodetector fabricated at 120 °C exhibited an optimized performance and a slowly declining response for each device depending on the wavelength derived from the effect of ZnO QDs. Correspondingly, the R_s_, D *, and EQE of the PD4 device were 374 μA W^−1^, 4.01 × 10^10^ Jones, and 0.1548% at 300 nm, respectively. As summarized in Table 3, the ZnO QD/(PEA)_2_PbI_4_ nanosheet hybrid photodetector PD4 possessed an outstanding performance among perovskite photodetectors [51,52,53,54,55,56,57,58,59], offering an approach for the fabrication of high-performance perovskite photodetectors with excellent stability.

## 4. Conclusions

In summary, the 0D ZnO quantum dot (QD)/2D (PEA)_2_PbI_4_ nanosheet hybrid photodetectors were fabricated via a facile ZnO QD chloroform antisolvent method under atmospheric conditions. Benefiting from the distinctive difference in polarity between the antisolvent and precursor solutions, the crystallization of the (PEA)_2_PbI_4_ thin films was effectively improved as a result of the accelerated nucleation site formation and grain growth rates. The uniform ornamentation of ZnO QDs on (PEA)_2_PbI_4_ nanosheets provided an in situ surface passivation for the surface defects as evidenced by the TRPL decay fitting, and the carrier transportation within the perovskite thin films was concurrently enhanced with the intensified charge separation induced by the well-balanced energy band levels. As a consequence, the photoelectric performance of ZnO QD/(PEA)_2_PbI_4_ nanosheet hybrid photodetectors sensitively developed with a variation of ZnO QD concentrations, resulting in excellent D * of 1.54 × 10^10^ Jones and I_on_/I_off_ ratio of 1040 for the device fabricated with the 0.097 mg/mL ZnO QD antisolvent. 

The annealing temperature-dependent behavior of photoconductive devices originated from the surface morphological evolution of the perovskite thin films, and the photodetector fabricated at 120 °C maintained a fast response speed within 100 ms and outstanding atmospheric stability after a 50-cycle test, offering a facile yet effective strategy for the ever-increasing demand for highly sensitive and long-term stable photodetection based on low-dimensional perovskites.

## Figures and Tables

**Table 1:** Summary of TRPL decay fitting characteristics for the photodetectors PD1 and PD4 based on the decay functions.

	PD1	PD4
τ_1_ (ns)	0.69	0.53
τ2 (ns)	3.10	3.27
τ3 (ns)	45.67	-
B_1_ (%)	53.52	84.82
B_2_ (%)	38.62	15.18
B_3_ (%)	7.87	-
τ_avg_ (ns)	5.16	0.94

**Table 2:** Summary of the Rs, D^*^, I_on_/I_off_ ratio, τ_rise_, and τ_fall_ of each photodetector at 10 V bias voltage and under 500 nm light illumination.

Photodetectors	R_s_ (μA/W)	D^*^ (×10^9^ Jones)	I_on_/I_off_ Ratio	τ_rise_ (ms)	τ_fall_ (ms)
PD1	14.21	1.32	78.3	95.8	106.8
PD2	100.73	9.48	564	69.1	46.7
PD3	142.40	13.50	809	126.7	53.0
PD4	143.94	15.40	1040	53.3	87.0
PD5	67.45	6.82	437	69.0	87.7
PD6	36.99	3.93	265	42.9	89.0
PD7	23.77	2.46	173	69.1	88.2
PD8	39.81	1.73	48.6	53.3	86.6
PD9	88.31	3.96	113	37.3	72.8
PD10	130.88	9.87	473	53.4	82.1
PD11	131.11	8.88	381	53.2	92.3

**Table 3:** Comparison of the photodetector performance created from different perovskite materials.

Materials	I_off_ (nA)	Bias (V)	I_on_/I_off_ Ratio	D^*^ (×10^9^ Jones)	Ref.
CH_3_NH_3_PbI_3_	1	1	3.05	900	[51]
CsPbBr3	0.488	2	1.7 × 10^6^	0.456	[52]
(PA)_2_(MA)Pb_2_I_7_	~0.1	10	>10^3^	29.2	[53]
MAPbI_3_	>10^2^	15	1100	12	[54]
(PEA)_2_PbI_4_, MoS_2_	~10^3^	3	500	8.09	[55]
BDAPbI4	>10^−2^	10	/	~1	[56]
MAPbI_3-x_Br_x_	4	4	<10	20	[57]
EA_4_Pb_3_Cl_10_	/	5	~10^4^	3.06	[58]
(PEA)_2_PbI_4_	~10^−3^	5	10.8	1070	[59]
(PEA)_2_PbI_4_, ZnO	4.37 × 10^−3^	10	1040	15.40	This Work

## Data Availability

The data is available upon reasonable request from the corresponding author.

## Supplementary Materials

The following supporting information can be downloaded at: https://www.mdpi.com/article/10.3390/nano12234217/s1, Figure S1: (a,b) Transmission electron microscope (TEM) images with different magnifications of ZnO QD decorated (PEA)_2_PBI_4_ nanosheets. (c) The selected area electron diffraction (SAED) pattern of the nanosheets. (d) Scanning electron microscope (SEM) images of the ZnO QD/(PEA)_2_PBI_4_ nanosheet sample blended with a proportion of 0.776 mg/mL ZnO QDs. Atomic force microscopy (AFM) images of the samples with different ZnO QD concentrations: (e) PD1 (0 mg/mL) and (f) PD4 (0.097 mg/mL); Figure S2: Tauc plot of the device blended with different proportions of ZnO QDs. Figure S3: (a) The plots of external quantum efficiency (EQE) for each device with different contents of ZnO QDs acquired at 500 nm. (b) The spectrum-dependent normalized detectivity (D *), (c) external quantum efficiency (EQE), and (d) I_on_/I_off_ ratio plots with different contents of ZnO QDs. Figure S4: The single-period responses of the devices fabricated at (a) 80 °C (PD9), (b) 100 °C (PD10), and (c) 140 °C (PD11), respectively.

## References

[1] Ghosh S., Kumar H., Mukhopadhyay B., Chang G.-E. (2021). Design and Modeling of High-Performance DBR-Based Resonant-Cavity-Enhanced GeSn Photodetector for Fiber-Optic Telecommunication Networks. IEEE Sens. J..

[2] Dong T., Simões J., Yang Z. (2020). Flexible Photodetector Based on 2D Materials: Processing, Architectures, and Applications. Adv. Mater. Interfaces.

[3] Bartolo-Perez C., Chandiparsi S., Mayet A.S., Cansizoglu H., Gao Y., Qarony W., AhAmed A., Wang S.-Y., Cherry S.R., Saif Islam M. (2021). Avalanche Photodetectors with Photon Trapping Structures for Biomedical Imaging Applications. Opt. Express.

[4] Zhang Y., Hu M., Wang Z. (2020). Enhanced Performances of P-Si/N-ZnO Self-Powered Photodetector by Interface State Modification and Pyro-Phototronic Effect. Nano Energy.

[5] Chen J., Ouyang W., Yang W., He J., Fang X. (2020). Recent Progress of Heterojunction Ultraviolet Photodetectors: Materials, Integrations, and Applications. Adv. Funct. Mater..

[6] Li J., Zhang G., Zhang Z., Li J., Uddin Z., Zheng Y., Shao Y., Yuan Y., Yang B. (2021). Defect Passivation via Additive Engineering to Improve Photodetection Performance in CsPbI_2_Br Perovskite Photodetectors. ACS Appl. Mater. Interfaces.

[7] Yu J., Liu G., Chen C., Li Y., Xu M., Wang T., Zhao G., Zhang L. (2020). Perovskite CsPbBr3 Crystals: Growth and Applications. J. Mater. Chem. C.

[8] Loi H., Cao J., Guo X., Liu C., Wang N., Song J., Tang G., Zhu Y., Yan F. (2020). Gradient 2D/3D Perovskite Films Prepared by Hot-Casting for Sensitive Photodetectors. Adv. Sci..

[9] Zhang Y., Li S., Li Z., Liu H., Liu X., Chen J., Fang X. (2020). High-Performance Two-Dimensional Perovskite ca_2_Nb_3_O_10_ UV Photodetectors. Nano Lett..

[10] Wang Y., Ma Z.-Z., Li Y., Zhang F., Chen X., Shi Z.-F. (2021). Low-Dimensional Phases Engineering for Improving the Emission Efficiency and Stability of Quasi-2D Perovskite Films*. Chin. Phys. B.

[11] Yue Y., Li M., Li H., Chai N., Dong Y., Li Z., Chen X., Wang X. (2022). One-Step Anti-Solvent Associated Method for High Performance Two-Dimensional Perovskite Photodetectors Fabrication at Low Temperature. Chem. Eng. J..

[12] Cheng B., Li T.-Y., Wei P.-C., Yin J., Ho K.-T., Retamal J.R.D., Mohammed O.F., He J.-H. (2018). Layer-Edge Device of Two-Dimensional Hybrid Perovskites. Nat. Commun..

[13] Malekshahi Byranvand M., Behboodi-Sadabad F., Alrhman Eliwi A., Trouillet V., Welle A., Ternes S., Hossain I.M., Khan M.R., Schwenzer J.A., Farooq A. (2020). Chemical Vapor Deposited Polymer Layer for Efficient Passivation of Planar Perovskite Solar Cells. J. Mater. Chem. A.

[14] Liang Y., Shang Q., Wei Q., Zhao L., Liu Z., Shi J., Zhong Y., Chen J., Gao Y., Li M. (2019). Lasing from Mechanically Exfoliated 2D Homologous Ruddlesden–Popper Perovskite Engineered by Inorganic Layer Thickness. Adv. Mater..

[15] Zanca C., Piazza V., Agnello S., Patella B., Ganci F., Aiello G., Piazza S., Sunseri C., Inguanta R. (2021). Controlled Solution-Based Fabrication of Perovskite Thin Films Directly on Conductive Substrate. Thin Solid Film..

[16] Jha S., Hasan M., Khakurel N., Ryan C.A., McMullen R., Mishra A., Malko A.V., Zakhidov A.A., Slinker J.D. (2022). Electrochemical Characterization of Halide Perovskites: Stability & Doping. Mater. Today Adv..

[17] Di Girolamo D., Dini D. (2022). Electrodeposition as a Versatile Preparative Tool for Perovskite Photovoltaics: Aspects of Metallization and Selective Contacts/Active Layer Formation. Sol. RRL.

[18] Swartwout R., Hoerantner M.T., Bulović V. (2019). Scalable Deposition Methods for Large-Area Production of Perovskite Thin Films. Energy Environ. Mater..

[19] Fang F., Wan Y., Li H., Fang S., Huang F., Zhou B., Jiang K., Tung V., Li L.-J., Shi Y. (2022). Two-Dimensional Cs_2_AgBiBr_6_/WS_2_ Heterostructure-Based Photodetector with Boosted Detectivity via Interfacial Engineering. ACS Nano.

[20] Subramanian A., Akram J., Hussain S., Chen J., Qasim K., Zhang W., Lei W. (2019). High-Performance Photodetector Based on a Graphene Quantum Dot/CH_3_NH_3_PbI_3_ Perovskite Hybrid. ACS Appl. Electron. Mater..

[21] Li H., Li Z., Liu S., Li M., Wen X., Lee J., Lin S., Li M.-Y., Lu H. (2022). High Performance Hybrid MXene Nanosheet/CsPbBr3 Quantum Dot Photodetectors with an Excellent Stability. J. Alloys Compd..

[22] Wu D., Wang Y., Ma N., Cao K., Zhang W., Chen J., Wang D., Gao Z., Xu F., Jiang K. (2019). Single-Crystal-like ZnO Mesoporous Spheres Derived from Metal Organic Framework Delivering High Electron Mobility for Enhanced Energy Conversion and Storage Performances. Electrochim. Acta.

[23] Shen K., Li X., Xu H., Wang M., Dai X., Guo J., Zhang T., Li S., Zou G., Choy K.-L. (2019). Enhanced Performance of ZnO Nanoparticle Decorated All-Inorganic CsPbBr_3_ Quantum Dot Photodetectors. J. Mater. Chem. A.

[24] Li Z., Yu X., Zhu Y., Liu S., Wen X., Lu H., Wang C., Li X., Li M.-Y., Yang Y. (2022). High Performance ZnO Quantum Dot (QD)/ Magnetron Sputtered ZnO Homojunction Ultraviolet Photodetectors. Appl. Surf. Sci..

[25] Li L., Liu J., Zeng M., Fu L. (2020). Space-Confined Growth of Metal Halide Perovskite Crystal Films. Nano Res..

[26] Huang F., Siffalovic P., Li B., Yang S., Zhang L., Nadazdy P., Cao G., Tian J. (2020). Controlled Crystallinity and Morphologies of 2D Ruddlesden-Popper Perovskite Films Grown without Anti-Solvent for Solar Cells. Chem. Eng. J..

[27] Jung M.H. (2021). Exploration of Two-Dimensional Perovskites Incorporating Methylammonium for High Performance Solar Cells. CrystEngComm.

[28] Singh A.K., Pal P., Gupta V., Yadav T.P., Gupta V., Singh S.P. (2018). Green Synthesis, Characterization and Antimicrobial Activity of Zinc Oxide Quantum Dots Using Eclipta Alba. Mater. Chem. Phys..

[29] Yang S., Niu W., Wang A.-L., Fan Z., Chen B., Tan C., Lu Q., Zhang H. (2017). Ultrathin Two-Dimensional Organic-Inorganic Hybrid Perovskite Nanosheets with Bright, Tunable Photoluminescence and High Stability. Angew. Chem. Int. Ed..

[30] Lan J., Lv J., Feng J. (2013). Identification of Chrome Pigments in Paints with Fourier Transform Infrared Spectroscopy (FTIR), Confocal Raman Microscopy, and Scanning Electron Microscope-Energy Dispersive Spectrometer. Environ. Forensics.

[31] Sánchez J.D.G., Messina S., Álvarez J.C., Nair P.K. (2022). Optical Absorption and Light-Generated Current Density in Chemically Deposited Antimony Sulfide Selenide Thin Films Used for Solar Cell Development. J. Mater. Sci. Mater. Electron..

[32] Hu R., Zhang Y., Paek S., Gao X.-X., Li X., Nazeeruddin M.K. (2020). Enhanced Stability of α-Phase FAPbI_3_ Perovskite Solar Cells by Insertion of 2D (PEA)_2_PbI_4_ Nanosheets. J. Mater. Chem. A.

[33] Adhikari N., Dubey A., Gaml E.A., Vaagensmith B., Reza K.M., Mabrouk S.A.A., Gu S., Zai J., Qian X., Qiao Q. (2016). Crystallization of a Perovskite Film for Higher Performance Solar Cells by Controlling Water Concentration in Methyl Ammonium Iodide Precursor Solution. Nanoscale.

[34] Tu Y., Xu Y., Li J., Hao Q., Liu X., Qi D., Bao C., He T., Gao F., Zhang W. (2020). Ultrathin Single-Crystalline 2D Perovskite Photoconductor for High-Performance Narrowband and Wide Linear Dynamic Range Photodetection. Small.

[35] Song J., Fang T., Li J., Xu L., Zhang F., Han B., Shan Q., Zeng H. (2018). Organic–Inorganic Hybrid Passivation Enables Perovskite QLEDs with an EQE of 16.48%. Adv. Mater..

[36] Chen H., Guo A., Zhu J., Cheng L., Wang Q. (2019). Tunable Photoluminescence of CsPbBr_3_ Perovskite Quantum Dots for Their Physical Research. Appl. Surf. Sci..

[37] Zhou L., Yu K., Yang F., Cong H., Wang N., Zheng J., Zuo Y., Li C., Cheng B., Wang Q. (2017). Insight into the Effect of Ligand-Exchange on Colloidal CsPbBr_3_ Perovskite Quantum Dot/Mesoporous-TiO_2_ Composite-Based Photodetectors: Much Faster Electron Injection. J. Mater. Chem. C.

[38] Liu H., Wang C., Liu D., Luo J. (2019). Neutral and Defect-Induced Exciton Annihilation in Defective Monolayer WS_2_. Nanoscale.

[39] Yang L., Wang Y., Xu H., Liu W., Zhang C., Wang C., Wang Z., Ma J., Liu Y. (2018). Color-Tunable ZnO/GaN Heterojunction LEDs Achieved by Coupling with Ag Nanowire Surface Plasmons. ACS Appl. Mater. Interfaces.

[40] Zhu L., Lu Q., Li C., Wang Y., Deng Z. (2021). Graded Interface Engineering of 3D/2D Halide Perovskite Solar Cells through Ultrathin (PEA)2PbI4 Nanosheets. Chin. Chem. Lett..

[41] Lee S.-W., Cha S.-H., Choi K.-J., Kang B.-H., Lee J.-S., Kim S.-W., Kim J.-S., Jeong H.-M., Gopalan S.-A., Kwon D.-H. (2016). Low Dark-Current, High Current-Gain of PVK/ZnO Nanoparticles Composite-Based UV Photodetector by PN-Heterojunction Control. Sensors.

[42] Yang Q., Guo X., Wang W., Zhang Y., Xu S., Lien D.H., Wang Z.L. (2010). Enhancing Sensitivity of a Single ZnO Micro-/Nanowire Photodetector by Piezo-Phototronic Effect. ACS Nano.

[43] Gong X., Tong M., Xia Y., Cai W., Moon J.S., Cao Y., Yu G., Shieh C.-L., Nilsson B., Heeger A.J. (2009). High-Detectivity Polymer Photodetectors with Spectral Response from 300 nm to 1450 nm. Science.

[44] Xiao H., Liang T., Xu M. (2019). Growth of Ultraflat PbI_2_ Nanoflakes by Solvent Evaporation Suppression for High-Performance UV Photodetectors. Small.

[45] Bi C., Shao Y., Yuan Y., Xiao Z., Wang C., Gao Y., Huang J. (2014). Understanding the Formation and Evolution of Interdiffusion Grown Organolead Halide Perovskite Thin Films by Thermal Annealing. J. Mater. Chem. A.

[46] Ma W., Yang C., Gong X., Lee K., Heeger A.J. (2005). Thermally Stable, Efficient Polymer Solar Cells with Nanoscale Control of the Interpenetrating Network Morphology. Adv. Funct. Mater..

[47] Hsu C.-J., Duan H.-S., Yang W., Zhou H., Yang Y. (2013). Benign Solutions and Innovative Sequential Annealing Processes for High Performance Cu2ZnSn(Se,S)4Photovoltaics. Adv. Energy Mater..

[48] Yu J.C., Kim D.W., Kim D.B., Jung E.D., Park J.H., Lee A.-Y., Lee B.R., Di Nuzzo D., Friend R.H., Song M.H. (2016). Improving the Stability and Performance of Perovskite Light-Emitting Diodes by Thermal Annealing Treatment. Adv. Mater..

[49] Venkataprasad Bhat S., Vivekchand S.R.C., Govindaraj A., Rao C.N.R. (2009). Photoluminescence and Photoconducting Properties of ZnO Nanoparticles. Solid State Commun..

[50] Yang L., Zhang Y., Ruan W., Zhao B., Xu W., Lombardi J.R. (2009). Improved Surface-Enhanced Raman Scattering Properties of TiO_2_Nanoparticles by Zn Dopant. J. Raman Spectrosc..

[51] Cao F., Tian W., Gu B., Ma Y., Lu H., Li L. (2017). High-Performance UV–Vis Photodetectors Based on Electrospun ZnO Nanofiber-Solution Processed Perovskite Hybrid Structures. Nano Res..

[52] Dong Y., Gu Y., Zou Y., Song J., Xu L., Li J., Xue J., Li X., Zeng H. (2016). Improving All-Inorganic Perovskite Photodetectors by Preferred Orientation and Plasmonic Effect. Small.

[53] Han S., Wang P., Zhang J., Liu X., Sun Z., Huang X., Li L., Ji C., Zhang W., Teng B. (2018). Exploring a Polar Two-Dimensional Multi-Layered Hybrid Perovskite of (C_5_H_11_NH_3_)_2_(CH_3_NH_3_)Pb_2_I_7_ for Ultrafast-Responding Photodetection. Laser Photonics Rev..

[54] Maculan G., Sheikh A.D., Abdelhady A.L., Saidaminov M.I., Haque M.A., Murali B., Alarousu E., Mohammed O.F., Wu T., Bakr O.M. (2015). CH_3_NH_3_PbCl_3_ Single Crystals: Inverse Temperature Crystallization and Visible-Blind UV-Photodetector. J. Phys. Chem. Lett..

[55] Fang C., Wang H., Shen Z., Shen H., Wang S., Ma J., Wang J., Luo H., Li D. (2019). High-Performance Photodetectors Based on Lead-Free 2D Ruddlesden–Popper Perovskite/MoS_2_ Heterostructures. ACS Appl. Mater. Interfaces.

[56] Zhang Y., Liu Y., Xu Z., Yang Z., Liu S. (2020). (Frank) 2D Perovskite Single Crystals with Suppressed Ion Migration for High-Performance Planar-Type Photodetectors. Small.

[57] Fang Y., Dong Q., Shao Y., Yuan Y., Huang J. (2015). Highly Narrowband Perovskite Single-Crystal Photodetectors Enabled by Surface-Charge Recombination. Nat. Photonics.

[58] Wang S., Li L., Weng W., Ji C., Liu X., Sun Z., Lin W., Hong M., Luo J. (2019). Trilayered Lead Chloride Perovskite Ferroelectric Affording Self-Powered Visible-Blind Ultraviolet Photodetection with Large Zero-Bias Photocurrent. J. Am. Chem. Soc..

[59] Lin C.-H., Cheng B., Li T.-Y., Retamal J.R.D., Wei T.-C., Fu H.-C., Fang X., He J.-H. (2018). Orthogonal Lithography for Halide Perovskite Optoelectronic Nanodevices. ACS Nano.

