# Supplementary material for: High Performance 0D ZnO Quantum Dot/2D (PEA)2PbI4 Nanosheet Hybrid Photodetectors Fabricated via a Facile Antisolvent Method
Source: Nanomaterials (Basel). 2022 Nov 27;12(23):4217. doi: 10.3390/nano12234217 (PMC9738548; doi:10.3390/nano12234217)
Supplement: Supplementary file 1 [file nanomaterials-12-04217-s001.zip › nanomaterials-2048899-supplementary.pdf]

nanomaterials

Supplementary Materials

High Performance 0D ZnO Quantum Dot/2D $(PEA)_2PbI_4$ Nanosheet Hybrid Photodetectors Fabricated via a Facile Antisolvent Method

Shijie Liu, Hao Li, Haifei Lu, Yanran Wang, Xiaoyan Wen, Shuo Deng, Ming-Yu Li *, Sisi Liu *, Cong Wang * and Xiao Li

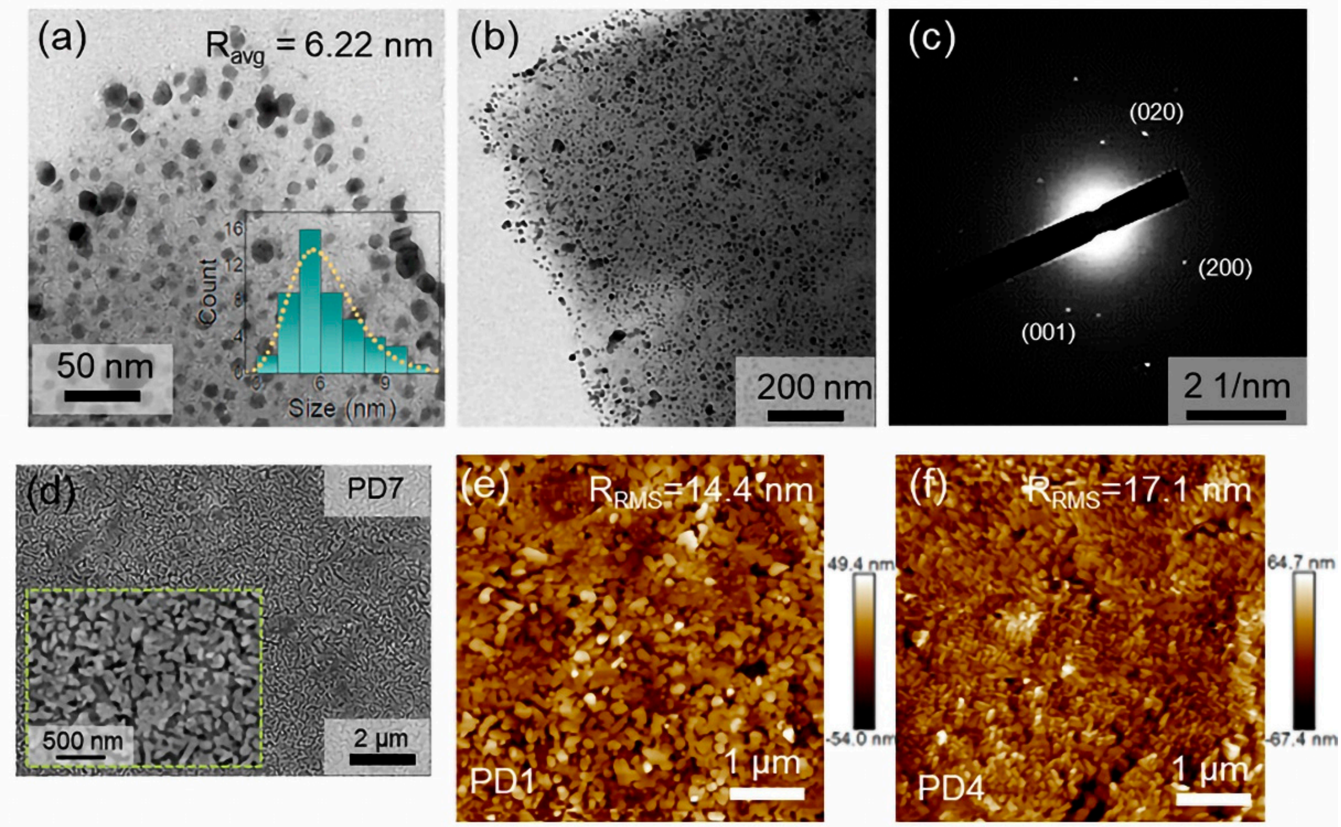

Figure S1. (**a**,**b**) Transmission electron microscope (TEM) images with different magnification of ZnO QD decorated $(PEA)_2PBI_4$ nanosheets. (**c**) The selected area electron diffraction (SAED) pattern of the nanosheets. (**d**) Scanning electron microscope (SEM) images of the ZnO QD / $(PEA)_2PBI_4$ nanosheet sample blended with a proportion of 0.776 mg/mL ZnO QDs. Atomic force microscopy (AFM) images of the samples with different ZnO QD concentrations: (**e**) PD1 (0 mg/mL) and (**f**) PD4 (0.097 mg/mL).

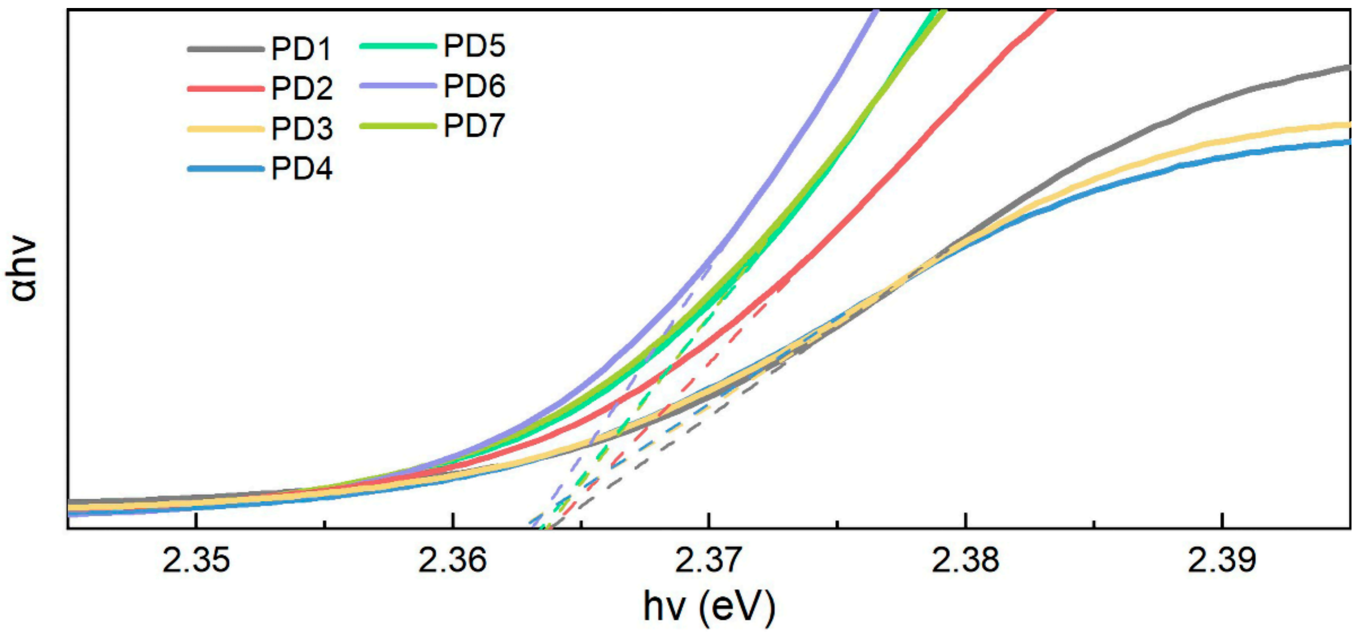

Figure S2. Tauc plot of the device blended with different proportions of ZnO QDs.

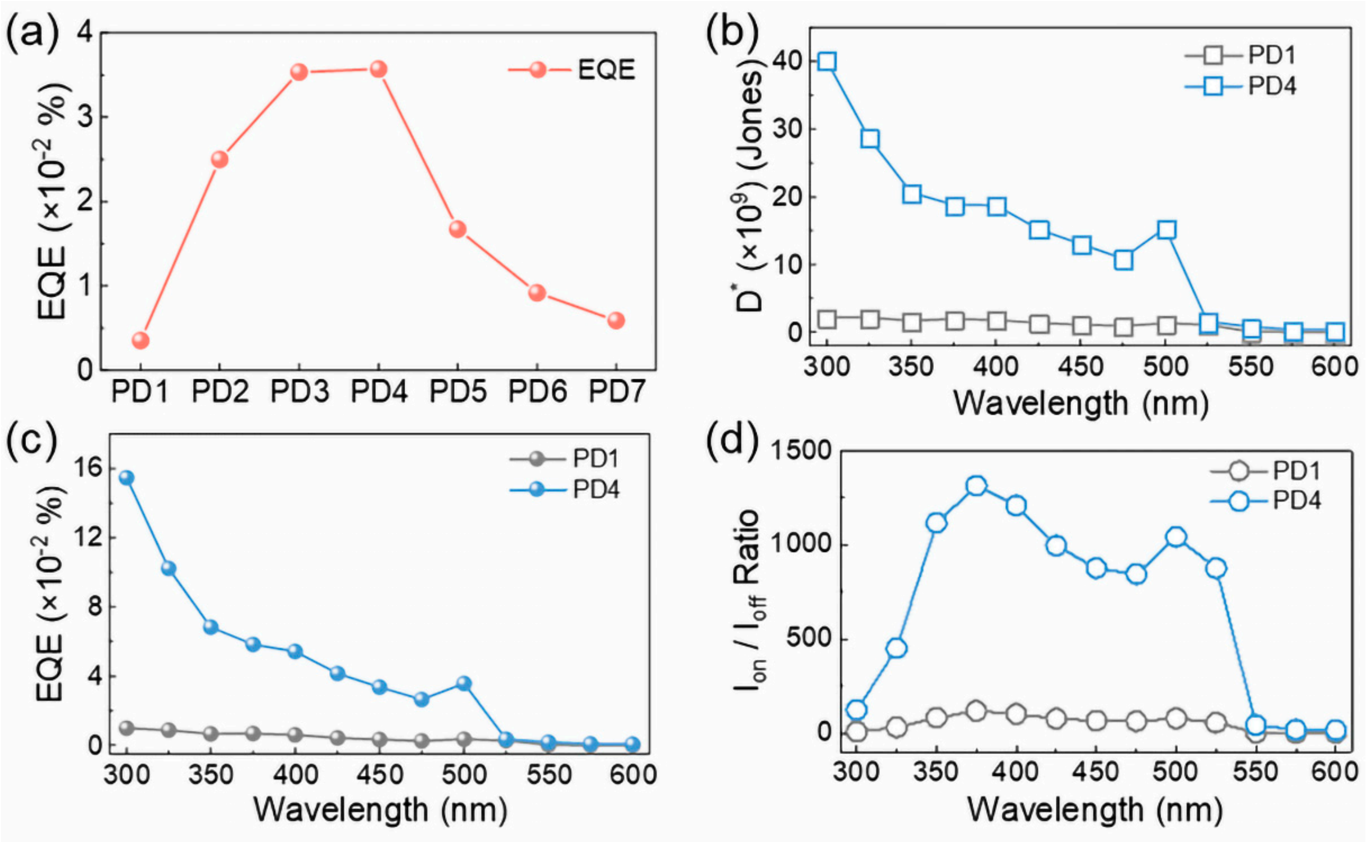

Figure S3. (**a**) The plots of external quantum efficiency (EQE) for each device with different contents of ZnO QDs acquired at 500 nm. (**b**) The spectrum dependent normalized detectivity (D^*), (**c**) external quantum efficiency (EQE), (**d**) I_{on} / I_{off} ratio plots with different contents of ZnO QDs.

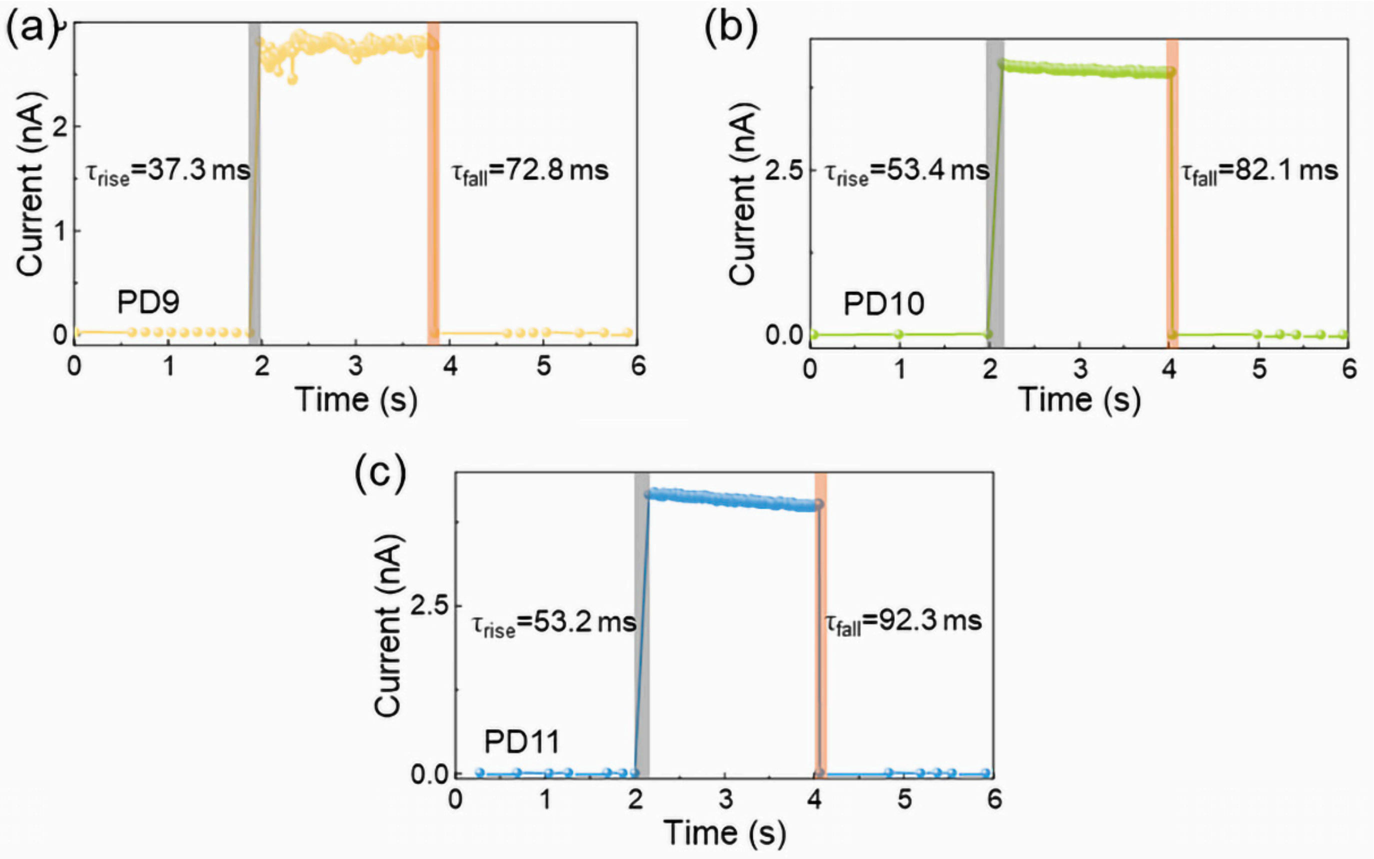

Figure S4. The single period response of the devices fabricated at (**a**) 80 °C (PD9), (**b**) 100 °C (PD10), (**c**) 140 °C (PD11), respectively.
